# Internet Hospitals in China: Cross-Sectional Survey

**DOI:** 10.2196/jmir.7854

**Published:** 2017-07-04

**Authors:** Xiaoxu Xie, Weimin Zhou, Lingyan Lin, Si Fan, Fen Lin, Long Wang, Tongjun Guo, Chuyang Ma, Jingkun Zhang, Yuan He, Yixin Chen

**Affiliations:** ^1^ Graduate School of Peking Union Medical College Beijing China; ^2^ National Research Institute for Health and Family Planning Beijing China; ^3^ Department of Urology, Jiangxi Cancer Hospital Nanchang China; ^4^ College of Electronic Information Science, Fujian Jiangxia University Fuzhou China; ^5^ Department of Obstetrics & Gynecology, The Second Affiliated Hospital of Nanchang University Nanchang China; ^6^ State Key Laboratory of Oncology in South China, Sun Yat-Sen University Cancer Center Guangzhou China; ^7^ Beijing Luhe International Academy Beijing China; ^8^ Sun Yat-sen University Guangzhou China; ^9^ Department of Computer Science and Engineering, Washington University in St. Louis St. Louis, MO United States

**Keywords:** eHealth, Internet, health care, medical informatics, China

## Abstract

**Background:**

The Internet hospital, an innovative approach to providing health care, is rapidly developing in China because it has the potential to provide widely accessible outpatient service delivery via Internet technologies. To date, China’s Internet hospitals have not been systematically investigated.

**Objective:**

The aim of this study was to describe the characteristics of China’s Internet hospitals, and to assess their health service capacity.

**Methods:**

We searched Baidu, the popular Chinese search engine, to identify Internet hospitals, using search terms such as “Internet hospital,” “web hospital,” or “cloud hospital.” All Internet hospitals in mainland China were eligible for inclusion if they were officially registered. Our search was carried out until March 31, 2017.

**Results:**

We identified 68 Internet hospitals, of which 43 have been put into use and 25 were under construction. Of the 43 established Internet hospitals, 13 (30%) were in the hospital informatization stage, 24 (56%) were in the Web ward stage, and 6 (14%) were in full Internet hospital stage. Patients accessed outpatient service delivery via website (74%, 32/43), app (42%, 18/43), or offline medical consultation facility (37%, 16/43) from the Internet hospital. Furthermore, 25 (58%) of the Internet hospitals asked doctors to deliver health services at a specific Web clinic, whereas 18 (42%) did not. The consulting methods included video chat (60%, 26/43), telephone (19%, 8/43), and graphic message (28%, 12/43); 13 (30%) Internet hospitals cannot be consulted online any more. Only 6 Internet hospitals were included in the coverage of health insurance. The median number of doctors available online was zero (interquartile range [IQR] 0 to 5; max 16,492). The median consultation fee per time was ¥20 (approximately US $2.90, IQR ¥0 to ¥200).

**Conclusions:**

Internet hospitals provide convenient outpatient service delivery. However, many of the Internet hospitals are not yet mature and are faced with various issues such as online doctor scarcity and the unavailability of health insurance coverage. China’s Internet hospitals are heading in the right direction to improve provision of health services, but much more remains to be done.

## Introduction

The Internet hospital, a new approach to outpatient health care, is the provision of health services via Internet technologies. Patients can now be at home or go to a local clinic. They can use a website or a smartphone app, and consult with a doctor based in a top-level hospital in a tier-one city [1]. The Internet hospital has become an emerging trend in China in recent years because it has the potential to provide widely accessible outpatient service delivery [1,2]. To date, China’s Internet hospitals have not been systematically investigated.

The rapid growth of the Internet and the increasing use of the Internet and mobile devices have contributed to a flourishing Internet medical services industry in China. The market for Chinese Internet medical services was 11.4 billion yuan (US $1.7 billion) in 2014 and 15.7 billion yuan in 2015, with an increase of 37.7% [3]. The population of Internet medical services users was 194.8 million in 2016, accounting for 26.6% of all Internet users [4].

In China, patients often have difficulty gaining access to appropriate health care [5]. In big-city top-flight hospitals, patients often queue overnight just to get a consultation lasting a few minutes [6]. The Internet hospital overcomes geographical obstacles and shatters time barriers, and as a result it has the capacity to address the difficulty of wait times by providing Chinese patients online access to skilled doctors.

The objective of this study was to provide an overview of the Internet hospitals in China as of March, 2017. We therefore conducted a cross-sectional study to describe the characteristics of China’s Internet hospitals, and to assess their health service capacity.

## Methods

### Selection of Internet Hospitals

We searched Baidu, the popular Chinese search engine, to identify Internet hospitals, using search terms such as “Internet hospital,” “web hospital,” or “cloud hospital.” The Internet hospitals mentioned in related news and reports were searched (up until March 31, 2017) using specific Internet hospital names. All Internet hospitals in mainland China were eligible for inclusion if they were officially registered. The inclusion criteria were as follows: simplified Chinese language of the Web page or app, service tailored toward mainland China (excluding Taiwan, Hong Kong, and Macau regions), and signed contracts with the local government or local hospital, officially acquiring the designations of Internet hospitals. We cross-referenced search results against a published list [7] and added any Internet hospitals that met the inclusion criteria. Two investigators (XX and LL) identified the Internet hospitals independently.

### Data Collection

After identifying the Internet hospital list, each Internet hospital was reviewed using information from the Internet hospital’s website and app (if available). An Excel (Microsoft) spreadsheet was used to record standard information of each Internet hospital. The data collected from each Internet hospital included the construction date; location; investor; consultation characteristics such as service object (provided service to patient or hospital), access method, consulting method (video chat, telephone, and graphic message; graphic message is a message with both text and graph), number of doctors available online (can deliver instant medical service), health insurance coverage, and consultation fee per time; and processes and stages.

We assessed the consultation characteristics of an Internet hospital by the official description, website, and smartphone app. The number of doctors available online for each Internet hospital was calculated as the median online doctors in 2 weeks. The consultation fee per time was the median fee of online doctors in one Internet hospital. If the Internet hospital had more than 100 doctors online simultaneously, we calculated the median of the top 100 doctors on the page.

Internet hospital processes included access to medical service, medical qualification, doctor source, medical service level, and medical service range. Access to medical services included online (defined as access to medical services via website or app) and offline (defined as access to medical services via local medical institutions) access. Medical qualification is an evaluation of whether the Internet hospital had or did not have a licensed medical qualification. Doctor sources included local hospitals, medical unions, district and nationwide. Medical service level was based on information (defined as appointment making, diagnosis or checkup report getting, and payment online); consultation (defined as remote diagnosis, electronic medical prescription, and health care management); and big data (defined as collection, analysis, and use of large amount of health care-related data and sharing data among hospitals).

Internet hospitals were divided into three stages (phases of development), including hospital informatization, Web ward, and full Internet hospital [7]. Those in the hospital informatization stage provide online appointments, getting reports, and paid services, and the doctors are from local hospitals or medical unions. Those in the Web ward stage provide purely online medical services, and the doctors are from local hospitals, medical unions, or regional hospitals. Full Internet hospitals provide an integration of online and offline medical services, and the doctors are from across the nation.

At least two of five investigators (XX, WZ, LL, SF, and FL) extracted the data independently. Any disagreements in abstracted data were resolved by discussion.

### Data Management and Analysis

Data management and analysis was performed using Microsoft Excel 2013. Nominal and ordinal data are presented using frequencies and percentages, bar charts, Venn diagrams, and statistical map. Skewed distributed continuous data are presented in percentile, median, and interquartile range.

## Results

### Internet Hospital Characteristics

**Figure 1:**
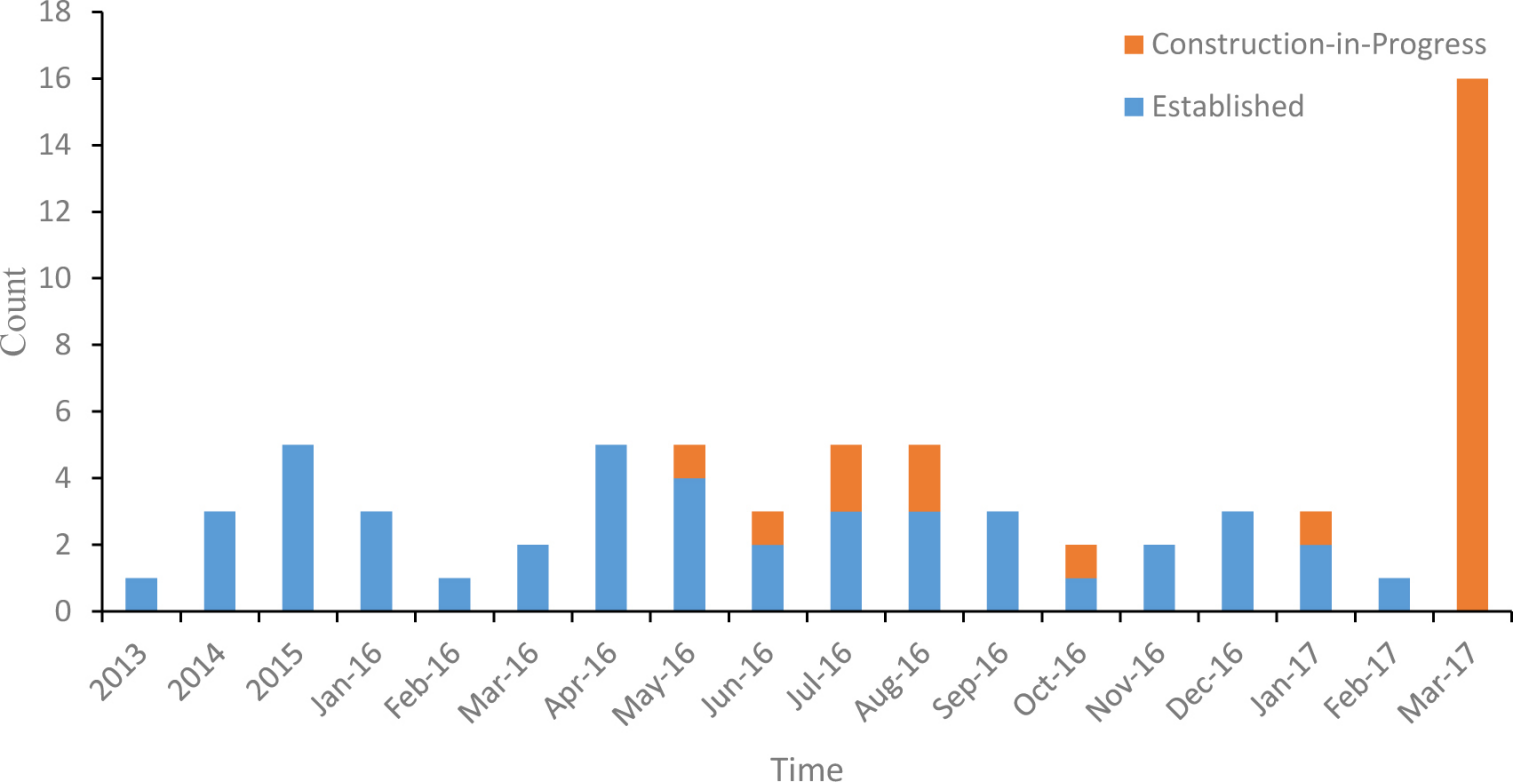
Construction date of Internet hospital in China.

**Figure 2:**
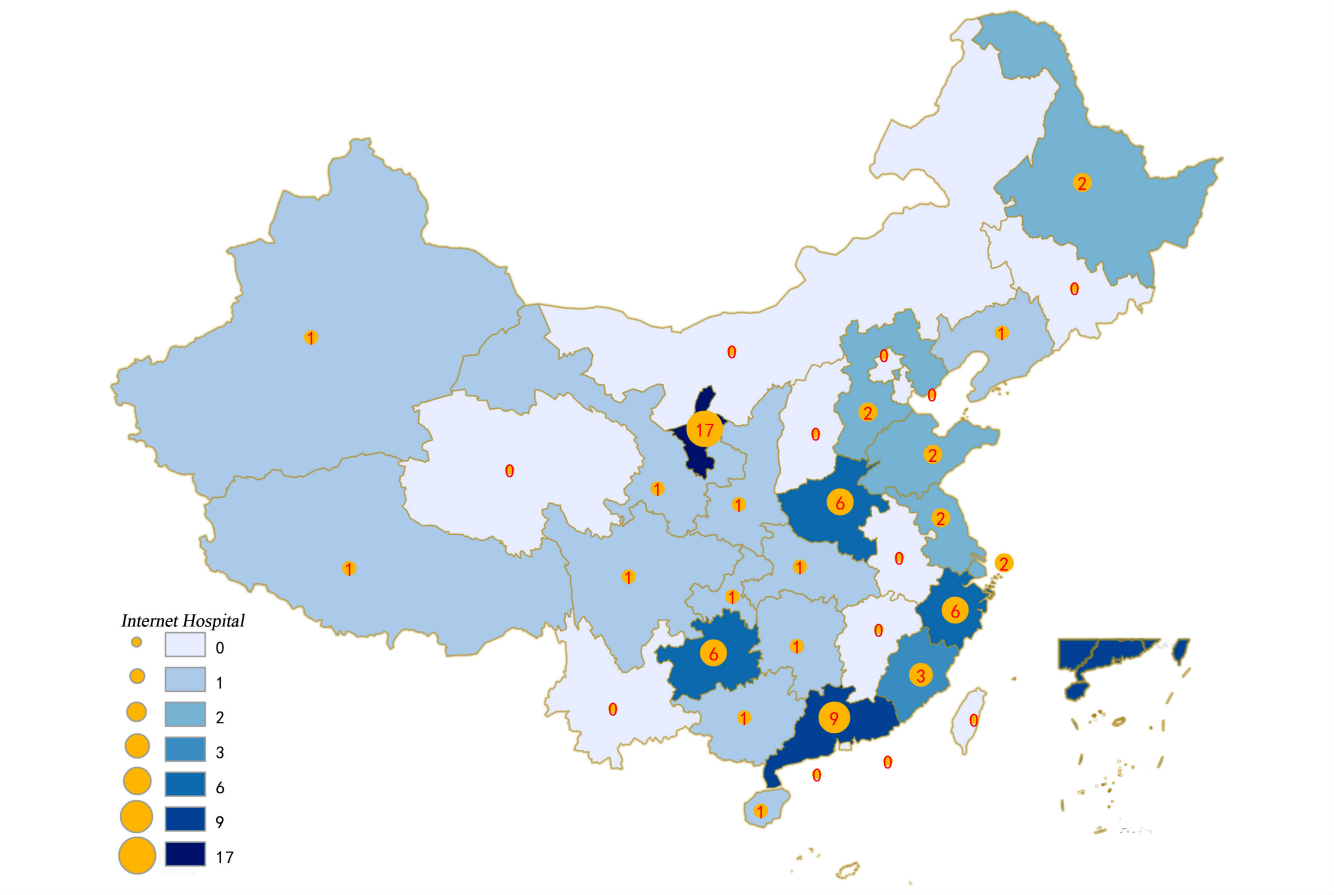
Spatial distributions of Internet hospitals in China.

We identified 68 Internet hospitals, of which 43 (63%) have been put into use and 25 (37%) were under construction. From inception until March 2017, 1 (1%), 3 (4%), 5 (7%), 39 (57%), and 20 (29%) Internet hospitals were launched in 2013, 2014, 2015, 2016, and 2017, respectively (Figure 1). The five leading provinces (Ningxia, Guangdong, Guizhou, Zhejiang, and Henan) had 44 Internet hospitals, accounting for 65% of all Internet hospitals in China (Figure 2). Most Internet hospitals were launched by Internet companies (72%, 49/68), followed by traditional hospitals (16%, 11/68), information technology (IT) service providers (6%, 4/68), pharmaceutical companies (3%, 2/68), and the government (3%, 2/68).

### Consultation

**Figure 3:**
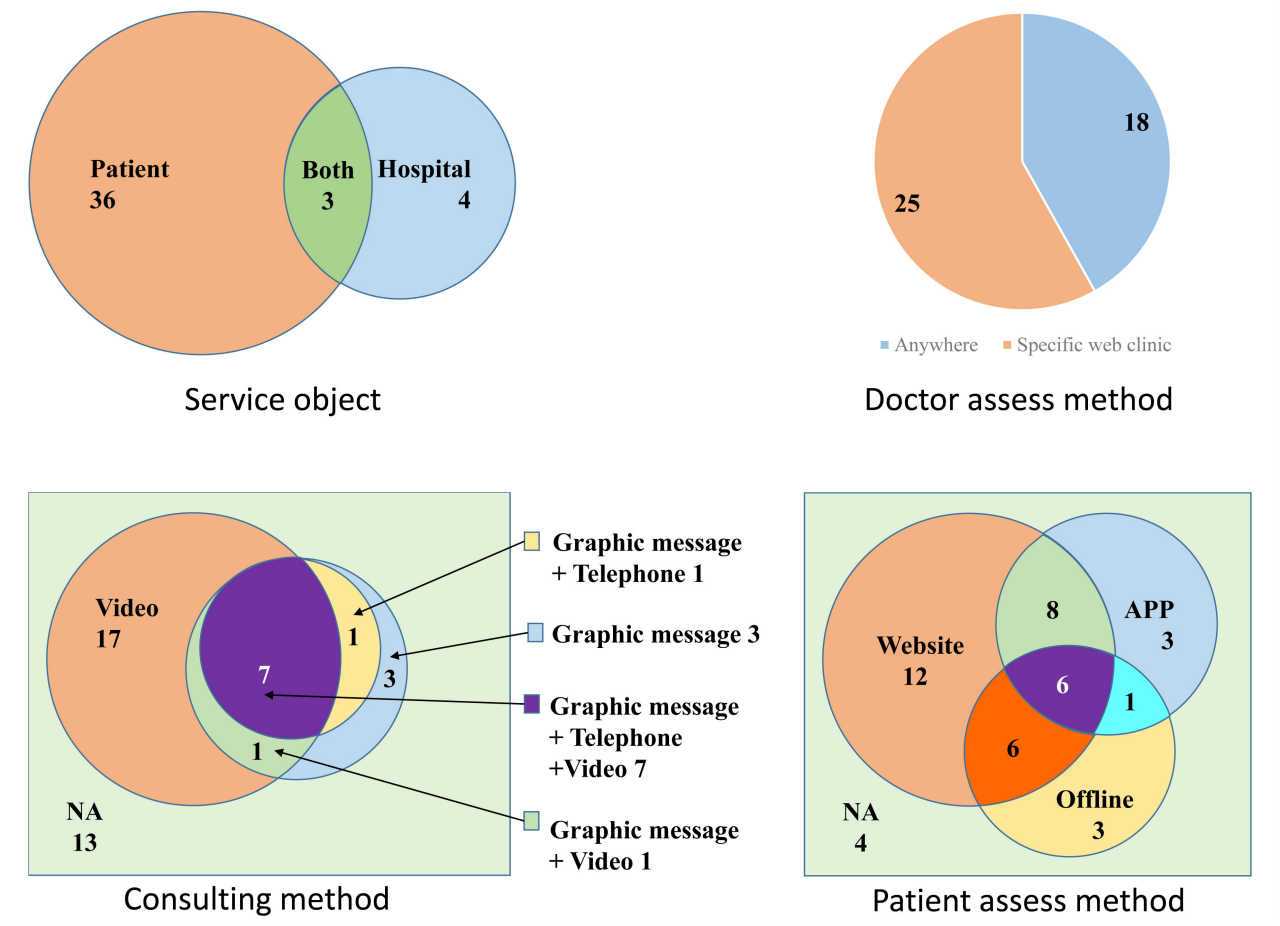
China’s Internet hospitals’ consultation characteristics.

In terms of the consulting characteristics of 43 established Internet hospitals, 3 (7%) of the Internet hospitals provided services to both patient and hospital, 36 (84%) provided services exclusively to patients, and 4 (9%) provided services exclusively to hospitals. Only 25 (58%) of the Internet hospitals asked doctors to deliver health services at a specific Web clinic, whereas 18 (42%) did not. Patients accessed outpatient service delivery via website (74%, 32/43), app (42%, 18/43), or offline medical consultation facility (37%, 16/43) from the Internet hospital. The consulting methods include video chat (60%, 26/43), telephone (19%, 8/43), and graphic message (28%, 12/43); 13 (30.2%) Internet hospitals cannot be consulted online any more (Figure 3). The median number of doctors available online was zero (interquartile [IQR] 0 to 5; max 16492). The median consultation fee per time was ¥20 (approximately US $2.90, IQR ¥0 to ¥200; Table 1).

**Table 1:** Number of doctors and consultation fee of Internet hospitals in China.

Variables	Minimum	P5^a^	P25	P50	P75	P95	Maximum
Number of doctors available online	0	0	0	0	5	224	16492
Consultation fee (yuan/time)	0	0	0	20	200	200	1000

^a^Px: xth percentile.

### Processes and Stages

Table 2 shows the processes and stages of the 43 established Internet hospitals. Of these, 13 (30%) were in hospital informatization stage, 24 (56%) were in Web ward stage, and 6 (14%) were in full Internet hospital stage. Additionally, 20 (47%) Internet hospitals could provide access to medical services through both online (website or smartphone app) and offline local medical institutions. Most Internet hospitals (95%, 41/43) had licensed medical qualification. Only 4 (9%) Internet hospitals had doctors from across the nation, 29 (67%) had doctors from local hospitals or medical unions, and 10 (23%) had doctors from regional hospitals. With regard to their medical service level, 5 (12%) Internet hospitals were at information-level, meaning, they helped patients register and get their diagnosis or the checkup report online. Furthermore, 34 (79%) Internet hospitals were at consultation-level and provided long-distance diagnosis, electronic medical prescription, and health care management; 4 (9%) Internet hospitals achieved big data-level, which entailed the collection, analysis, use of large amounts of healthcare–related data, and the of sharing of data among hospitals; and 30 (70%) Internet hospitals could distribute drugs to patients’ homes through cooperative pharmaceutical companies. Only 6 (14%) Internet hospitals were included in the coverage of health insurance.

**Table 2:** Processes and stages of Internet hospitals in China (N=43).

Characteristics		n (%)
**Stages**		
	Hospital informatization	13 (30)
	Web ward	24 (56)
	Full Internet hospital	6 (14)
**Access to medical service**		
	Online or offline	23 (53)
	Online and offline	20 (47)
**Medical qualification**		
	No	2 (5)
	Yes	41 (95)
**Doctor source**		
	Local hospital or medical union	29 (67)
	District	10 (23)
	Nationwide	4 (9)
**Medical service level**		
	Information	5 (12)
	Consultation	34 (79)
	Big data	4 (9)
**Medical service range**		
	Health care	13 (30)
	Health care + drug delivery or medical insurance	24 (56)
	Health care + drug delivery + medical insurance	6 (14)

## Discussion

### Statement of Principal Findings

This cross-sectional study provided an overview of China’s Internet hospital in March 2017.

Internet hospitals in China show an emerging trend and are clustered in the city with local policy support. Internet hospitals provide convenient outpatient service delivery through video chat, telephone, and graphic message. However, many of the Internet hospitals are not yet mature and are faced with various issues such as online doctor scarcity and the unavailability of health insurance coverage.

### A Surge in 2016

A key insight from our study is that there has been an emerging trend of Internet hospitals in China in recent years. Despite this, the first officially sanctioned Internet hospital in China was founded in October, 2014 [1]; only 9 Internet hospitals were launched by 2015. In 2016, however, a multitude of Internet hospitals were established in China, with approximately 39 newly-built Internet hospitals in the country. Furthermore, 20 Internet hospitals were launched by March 2017.

The emergence of Internet hospitals is partly due to the integration of offline businesses with online commerce, both in line with the current national efforts to promote the classification of treatment policy and the current market ecology [3,8,9]. On the other hand, the traditional medical resources’ inability to meet the needs of the public medical services also contributed to the development of Internet hospitals [1,7,10].

### Matthew Effect

We found there was a Matthew effect, especially with medical resources concentrated in a few leading Internet hospitals. Our findings show that the leading Internet hospital had about 16,500 doctors available online, but on the other hand, approximately 75% of Internet hospitals had no more than 5 doctors available online. One possibility is that in the leading Internet hospitals, which are sponsored by Internet enterprises, the consultation price can be set by the doctors themselves; this is approximately 200 yuan, generally far surpassing the traditional public hospitals’ standard rate of 5 to 20 yuan, and thus a major incentive for the doctors [9]. Another possibility is that the leading Internet hospital has a total of 26,000 licensed doctors in more than 2400 hospitals across China, and has branches in 19 cities and provinces including Beijing, Guangdong, Henan, Sichuan, Gansu, Guizhou, and Ningxia [11]. However, most Internet hospitals only have doctors from their own hospital or medical union.

### Processes and Stages

Another key finding from our study was that, although Internet hospitals help in improving the quality of medical services, many of the Internet hospitals are not yet mature and are faced with various issues such as online doctor scarcity and not being covered by health insurance. There is a shortage of doctors in China because the demand for medical services in the country has far outstripped supply [12,13]. Furthermore, the shortages are exacerbated by a high turnover of staff caused by heavy workloads, deteriorating doctor-patient relationships, and a resulting increase in work-related stress [12,14,15]. Those systemic issues should be resolved by the government to provide a positive medical environment [13].

The Internet hospital is a medical development model that includes health education, medical information, electronic health records, disease risk assessment, online disease counseling, electronic prescription, telemedicine, and remote treatment and rehabilitation with the Internet as a carrier and technical means [16]. However, most Internet hospitals remain at the hospital informatization stage or Web ward stage. The final stage of an Internet hospital should be a smart hospital, which entails providing big data, artificial intelligence, and precision medicine. China’s Internet hospitals are heading in the right direction to improve how health service is effectively provided, but much more remains to be done.

### Policy Support

To implement the ambitious strategy that China is now rolling out to improve its health system [17,18], several key challenges need to be met. The challenges are improvement of the quality of care, and enhancement of equity, including addressing disparities among China’s diverse regions. The Internet hospital has the potential to resolve part of these challenges [1,5]. The authorities encourage hospitals to offer patients more accessible medical services via the Internet according to a guideline released by the State Council in July 2015 [8].

Internet hospitals are clustered in the city with local policy support, including Internet medical services fees, health insurance, and other issues. Our study found that the five leading provinces had 44 Internet hospitals, accounting for 64.7% of all Internet hospitals in China. China’s first Internet hospital base was established on March 19, 2017, in the city of Yinchuan, capital of northwest China’s Ningxia Hui Autonomous Region. To make Internet hospitals a reality, Yinchuan city has come out with China’s first rules on the management of Internet hospitals and built a complete supervision and management system. Yinchuan city government will go on to link the Internet hospitals with the medical insurance system in 2017. Thus, 17 nationally known Internet medical service institutions signed contracts with the Yinchuan city government to establish Internet hospitals [19]. Other cities or provinces also provide policy support to Internet hospitals [20,21]; this bodes well for the economics of Internet hospitals.

### Limitations

A limitation of this study is that we had no related individual doctor or patient data, the use of which would have provided greater detail about health worker characteristics and outpatient service characteristics, allowing us to evaluate the health service capacity, medical resources, and patient satisfaction of Internet hospitals. A further limitation was that this study focused merely on Internet hospitals without traditional hospitals as controls; therefore, we cannot compare the advantages and disadvantages between them. These aspects should be investigated in future studies.

### Prospective

China’s top-level hospitals have continually invested in upgrading their IT infrastructure, and a lot of Internet companies such as We Doctor, Alibaba, and Tencent are involved in this industry [3,22]. Combined with the surge in use of smartphones, mobile Internet, and big investments in innovation [23], the Internet hospital has a promising future in China.

The Internet system gives patients in rural and remote areas better access to services and helps achieve the integration of online and offline medical services. China’s enthusiastic adoption of innovative eHealth systems could encourage the rest of the world to make the Internet an integral part of their medical care. With the Internet increasingly available across the globe, the innovations and experiences of China’s Internet hospital will be helpful and influential for any developing country whose medical resources are clustered in their big cities.

### Conclusions

The Internet hospital in China is a large and continuously growing market. By March 2017, it was apparent that although the Internet has the potential to overcome some of the challenges due to the rapid changing environment of health care needs, reform, and provision in China, many of the Internet hospitals had not yet matured, and faced various issues, such as online doctor scarcity and the unavailability of health insurance coverage.

## Abbreviations

IT: information technology
IQR: interquartile range
